# Relative impacts of fishing and eutrophication on coastal fish assessed by comparing a no-take area with an environmental gradient

**DOI:** 10.1007/s13280-018-1133-9

**Published:** 2018-12-06

**Authors:** Lena Bergström, Martin Karlsson, Ulf Bergström, Leif Pihl, Patrik Kraufvelin

**Affiliations:** 1 Department of Aquatic Resources, Institute of Coastal Research, Swedish University of Agricultural Sciences, Skolgatan 6, 742 42 Öregrund, Sweden; 2 University of Gothenburg, Kristineberg 566, 451 78 Fiskebäckskil, Sweden

**Keywords:** Coastal fish, Environmental management, Coastal ecology, Eutrophication, No-take areas, Status indicator

## Abstract

Understanding the relative impacts of pressures on coastal ecosystems is central for implementing relevant measures to reach environmental management objectives. Here, survey data on the species and size composition of coastal fish are evaluated in relation to fishing and eutrophication, by comparing a long-standing no-take area to an environmental gradient in the Baltic Sea. The no-take area represents an intermediate eutrophication level, but the species composition resembles that seen at low eutrophication in areas with fishing. The catch biomass of piscivores is 2–3 times higher in the no-take area than in the other areas, while the biomass of Cyprinids, generally benefitted by eutrophication, corresponds to that of areas with low eutrophication. The results support that fishing may generate eutrophication-like effects, and, conversely, that no-take areas may contribute to improving environmental status in impacted areas by enhancing piscivores, which in turn may contribute to further improvement in the food web.

**Electronic supplementary material:**

The online version of this article (10.1007/s13280-018-1133-9) contains supplementary material, which is available to authorized users.

## Introduction

Coastal fish provide important services to humans, such as food production and recreation, but are also recognized as central to ecosystem function (Holmlund and Hammer 1999). In particular, the species composition and size structure of fish are seen to contribute to regulating ecosystem services of relevance for conservation and environmental management. For example, large predatory fish species are not only directly valuable for fishing, but may also have a significant role in buffering negative effects of eutrophication and contributing to the resilience of coastal ecosystems (Eriksson et al. 2009; Sieben et al. 2011; Östman et al. 2016). Smaller mesopredatory species, i.e., benthivorous and planktivorous species, in turn, often have the opposite role and may accentuate such eutrophication effects (Bergström et al. 2015; Donadi et al. 2017).

Impacts on coastal ecosystems from human activities are, however, long-lasting and increasing today, with associated negative effects on the environmental status of species and habitats (Airoldi and Beck 2007; Andersen et al. 2015), and there is a continued need to protect biodiversity and enhance restoration measures. This issue is highly relevant also for coastal fish in the Baltic Sea (Bergström et al. 2016a; Kraufvelin et al. 2018). However, management may often be hampered by limited knowledge on the relative extent to which different human-induced pressures are affecting the environmental status.

This study aims at exploring the relative impacts on coastal fish from pressures exerted by fishing and eutrophication in a coastal region of the Baltic Sea. Although coastal environments are affected by numerous factors, these two factors are identified as having prevailing impact on the Baltic Sea environment, as well as in many other marine regions (Dayton et al. 1995; Uusitalo et al. 2016). Fishing is globally known to cause, for example, changes in species composition, truncated size structures, and habitat alterations (Jennings and Kaiser 1998), as also seen in the Baltic Sea. In offshore areas of the Baltic Sea, extensive fishing has been connected to dramatic declines in target species, especially of larger fish, and changes in food web structure (Casini et al. 2009; Svedäng and Hornborg 2017). The level of commercial fishing in coastal areas is considerably lower in comparison, but recreational fishing may on the other hand be substantial. Although associated with a relatively high level of uncertainty, it has been estimated that recreational fishing at the Swedish coast removes 5–20 times the biomass caught in coastal commercial fisheries for some central species (Karlsson et al. 2015; Hansson et al. 2017). Eutrophication, in turn, is identified as a long-lasting pressure on the Baltic Sea, affecting coastal as well as open sea areas, and entailing a continued need of measures to improve the situation (HELCOM 2018a). However, there may be significant spatial variation in the level of eutrophication, even among adjacent coastal areas, depending on local differences in nutrient inputs from land and levels of water exchange. Typically, enclosed areas under direct influence from land runoff are the most affected, but in some areas, contributions from the open sea may be important (Bryhn et al. 2017).

Changes in coastal fish have been widely observed in the Baltic Sea, and several studies have also assessed the relationships of selected fish species and species groups to environmental pressures (see references in Kraufvelin et al. 2018). For example, species within the taxonomic family Cyprinidae and pikeperch (*Sander lucioperca*), which are adapted to turbid conditions, are known to gain competitive advantage under eutrophication, while species dependent on clear water habitats, such as perch (*Perca fluviatilis*), are disadvantaged (Sandström and Karås 2002; Bergström et al. 2013; Snickars et al. 2015). Evidence is more scarce regarding responses to fishing in coastal areas, although a deterioration of target species has been seen when comparing areas with intense fishing to those with little or no fishing (Mustamäki et al. 2014), and comparing time periods of historically lower and higher fishing pressures (Eero 2004). The scarcity of knowledge is partly due to a lack of information on prevailing total levels of fishing pressure in coastal areas, especially regarding recreational fishing (Hyder et al. 2018), data that would be needed at a high spatial resolution to allow for studying pressure–response relationships. This aspect is particularly important as many coastal fish species have a local population structure and are likely to be strongly affected by local pressures (Saulamo and Neuman 2002; Östman et al. 2017a). In addition, many species of coastal fish are clearly responsive to climate changes (Olsson et al. 2012), making it difficult to verify the relative importance of different pressures in temporal studies.

Here, we evaluate the relative influence of fishing and eutrophication on coastal fish assemblages, using a field survey that compares a long-standing no-take area to sites along a eutrophication gradient. Although relatively small in size, the no-take area is exceptional for northern temperate waters, since long-term fishing closures as well as empirical evaluations of their effects are still remarkably scarce (see, e.g., Fenberg et al. 2012). We compare the no-take area with nearby areas representing a range of environmental conditions, with focus on different levels of eutrophication. Our aim is to clarify which impacts these two pressures may have on the composition of local fish assemblages, in terms of abundance, biomass, and size of key species. The results are intended to support the current need to develop management measures towards reaching environmental objectives for coastal areas.

## Materials and methods

Coastal fish in the brackish Baltic Sea include both freshwater and marine species and are here defined as assemblages of fish species which spend a main part of their life cycle in shallow (< 10 m) nearshore waters. The species of freshwater origin are well adapted to the brackish water environment and use coastal or adjacent freshwater areas for spawning and nursery. The marine species are either local residents or migrate to the coastal zone during certain life stages, such as for feeding or spawning. The study took place during the warmest time of the year, when coastal freshwater species predominate, and marine species are less abundant in the investigated shallow nearshore waters (Olsson et al. 2012).

### Characterization of the study areas

**Fig. 1:**
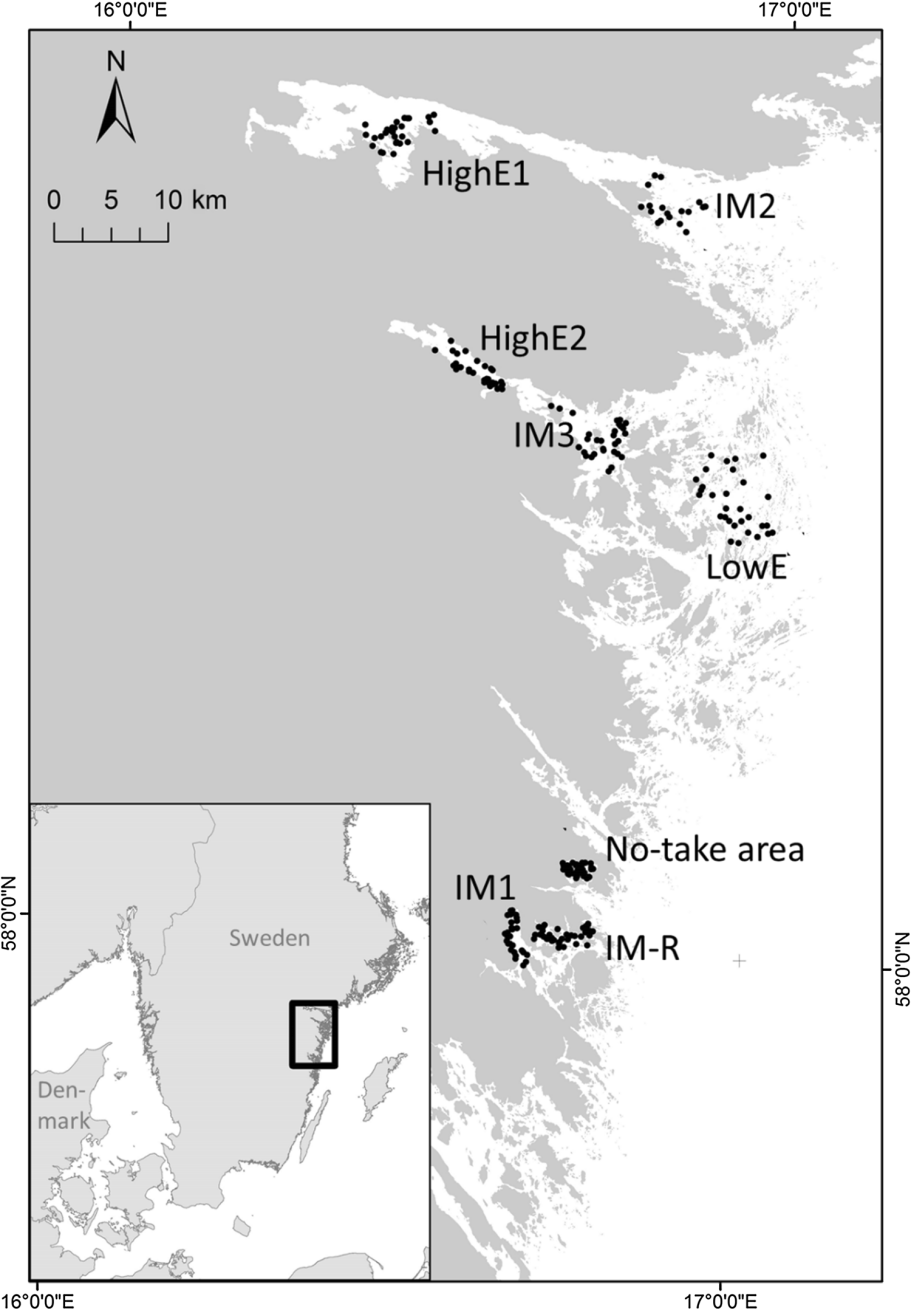
Positions of the sampled areas in the central Baltic Sea

The study was conducted in a topographically complex archipelago region of the Baltic Sea coast, covering eight areas (Fig. 1). One of the areas is a no-take area, where there has been virtually no fishing over the past 30 years. Together, the areas represent the major gradients in environmental variables found in the archipelago, such as salinity and wave exposure, as well as pronounced differences in the level of eutrophication (More details provided below). The focus of the study was on differences among areas, but it also enabled comparing this spatial variability with the range in temporal variability in one of the areas, which is a designated area for long-term monitoring of coastal fish.

The fishing closure was first implemented for nature protection in 1979. A limited artisanal fishery was allowed until the early 2000s when the area was completely closed (Bergström et al. 2016c). For the other areas, detailed information on fishing pressure is not available, but they are all known to be fished. All areas are included in the ICES spatial management unit SD27, for which total fish catches were recently estimated to 84 kg/km^2^ annually for perch (*Perca fluviatilis*) and 130 kg/km^2^ for pike (*Esox lucius*) for areas < 10 m deep in year 2010 (Hansson et al. 2017). These figures are based on commercial landing statistics and recreational landings assessed by a national questionnaire. Like in other parts along the Swedish coast, recreational catches constitute the majority of the total catches. The reported levels correspond to intermediate or high levels at a Baltic Sea scale. For comparison, median catches along the Baltic Sea coastline were estimated to 29–320 kg/km^2^ for perch (ICES SD24–32), and 3–190 kg/m^2^ for pike (Hansson et al. 2017). The study area is also important for pikeperch fishing, with catches around one-third of those of pike (Karlsson et al. 2015; Hansson et al. 2017).

The eutrophication level was represented by water clarity (Secchi depth), which is a main indicator of eutrophication in the Baltic Sea (HELCOM 2018a). Even though several ways of measuring eutrophication can be considered, Secchi depth is identified as a key indicator of habitat conditions for coastal fish in relation to eutrophication (Bergström et al. 2013), and provides a direct connection to how fish may be most closely impacted. As opposed to primary producers and first-order consumers, fish are not primarily affected by changes in productivity per se, but rather by habitat-related effects, for example, by changes in light conditions affecting foraging and predator escape behavior, or by changes in vegetation affecting spawning and nursery habitats. Further, Secchi depth is suitable for spatial analysis, which is in focus here, as measurements can be obtained at the level of each station.

**Fig. 2:**
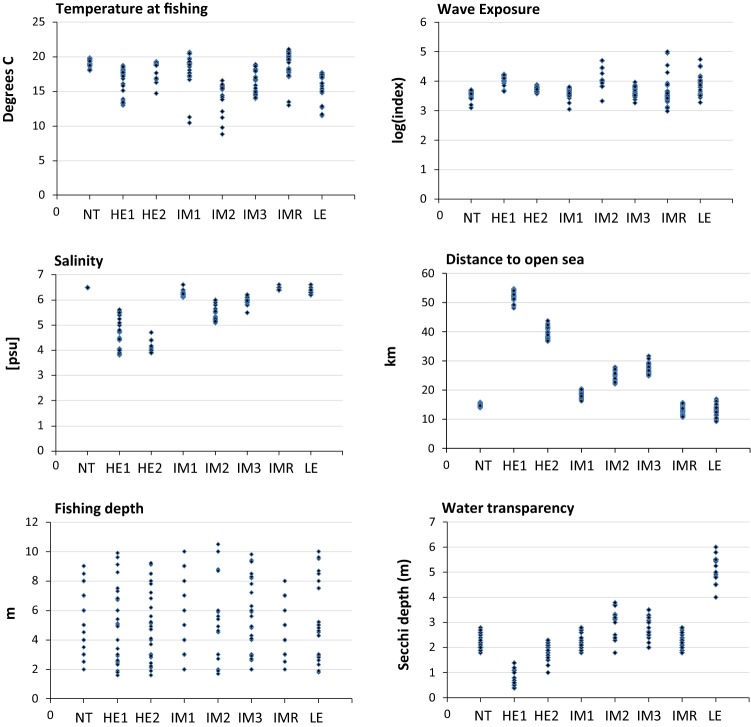
Range of values for the potential explanatory environmental variables in the studied areas. Area names and number of stations sampled: No-take area (NT, *N* = 35), Areas of highest eutrophication level (HE1, *N* = 30; HE2, *N* = 29), Intermediate eutrophication level (IM1, *N* = 30; IM2, *N* = 18; IM3, *N* = 30, IM-R; *N* = 38), Low eutrophication level (LE, *N* = 30). For more detailed statistics, see Table S1

For seven of the areas, the level of eutrophication was additionally characterized using hydrochemical monitoring data, collected in accordance with national standards for environmental monitoring. Water samples were taken at the surface at three sites within each area during June, July, and August at the year of fishing (Pihl et al. 2015). The samples represent the total nitrogen and phosphorus, as well as chlorophyll *a*, present in the water column during the growth season, with the main expected sources of nutrients being land-based runoff and influence from nutrient-enriched open sea water. By these more detailed data, two areas were characterized as representing the highest level of eutrophication (HighE1–2), based on high total nitrogen and chlorophyll *a* (and low water clarity; Table 1). One area in the outer archipelago was characterized as representing a low level of eutrophication (LowE), and remaining areas represented intermediate levels, including the long-term monitoring area (IM1–3, IM-R). Corresponding data were not available for the no-take area. The classification of the no-take area was made by comparing measurements of Secchi depth during fishing, which showed similar levels to the two most adjacent other areas (IM1, IM-R; Fig. 2, Table S1). An intermediate level of eutrophication in the no-take area was also supported by a similar salinity as in IM-R, suggesting a high level of water exchange between these adjacent water bodies, and likely similar nutrient levels (Bryhn et al. 2017).

**Table 1:**
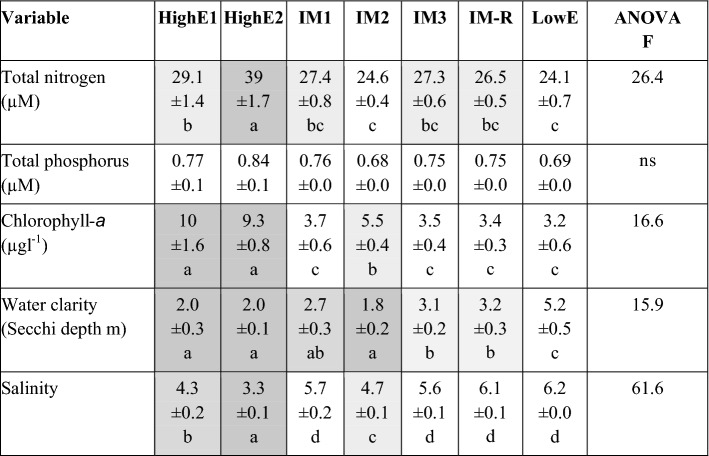
Characterization with respect to level of eutrophication of areas with available hydrochemical monitoring data. Letters and color shades identify discrete groups based on a posteriori SNK-tests, for variables with significant overall differences according to ANOVA (*p* < 0.01). *D.f.* = 6, 56. Values show the mean ± SE. For location of subareas, see Fig. 1

In addition, the areas varied in several environmental factors which may influence the species composition of coastal fish, such as salinity, distance to open sea, and wave exposure (Fig. 2, see also below). Most noticeably, the areas characterized as having highest eutrophication level had the lowest salinity, which reflects that a large pool of nutrients are carried to the sea via freshwater outflows (see also section on analyses).

### Fish surveys

Fish were surveyed using Nordic coastal multimesh gill nets applying standardized methodology. The nets are composed of nine panels of 5 m length with mesh sizes between 10 and 60 mm (total net length 45 m and depth 1.8 m). At least 30 stations were fished per area, with ten stations randomized within each of the depth strata 0–3 m, 3–6 m, and 6–10 m during 29 July–22 August 2013. The stations were located on soft bottom and mixed sediments. Vegetation cover was generally low, but was relatively highest in the shallowest stratum which allowed sufficient light conditions. All stations were fished overnight. Hydrographical profiles of the water column were examined to verify that nets were set above the thermocline. Catches were registered as number of individuals per species and cm length group. Weights were recorded by species and mesh size for the majority of the catch. In the statistical analyses, information on weights for the total catch was based on empirically determined length–weight relationships as applied in the national coastal fish database (www.slu.se/kul), and verified against 306 data points for which both direct and estimated weights were available (Pearson correlation coefficient *r* > 0.99).

Environmental variables such as water depth and temperature at the bottom were recorded at each station, as these may influence the catches (Bergström et al., 2016b), and so were salinity and water transparency (Secchi depth; Fig. 2, Table S1). Wave exposure was estimated in GIS using the simplified wave model index (SWM), which combines fetch calculations with wind conditions and accounts for wave refraction and diffraction (Isaeus 2004). Distance to open sea was quantified as the shortest waterway distance between each sampling station and the open sea, as defined by a geographical line connecting the outmost islands along the Swedish coast.

### Analyses

#### Multivariate analyses

The overall variability in species composition was assessed by multivariate analyses, using principal coordinates analysis (PCO) based on Bray–Curtis similarities after square root transformation (Anderson et al. 2015). The analyses were first performed based on data per fishing station, and one-way PERMANOVAs with pairwise a posteriori assessments were employed to assess differences in species composition among areas. Subsequently, the PCOs were re-run using average values for each area, as supported by the PERMANOVA results. In this part of the analyses, characteristic species for different areas were identified by examining the multiple correlations of fish species with the obtained PCO-axes, and vectors representing these species were projected on the PCO biplot. In order to compare the level of spatial variability with interannual variability, data covering the years 2002–2013 from the long-term monitoring area (IM-R) were also included (corresponding to all years for which data with the same monitoring methodology were available). All multivariate analyses were performed in parallel for data on biomass and abundance. The analyses were run in PRIMER 6.0 with PERMANOVA (Anderson 2017).

#### Univariate models

Further, general linear models (GLMs) were run for selected univariate metrics (Zuur et al. 2007) to evaluate the relationship of the response metrics to natural environmental variables (covariates), to a variable representing level of eutrophication (“Secchi depth”), and to remaining differences among areas in order to evaluate potential effects of the no-take area.

Following examination of variance inflation factors (VIF), the following variables, out of the ones sampled (Fig. 2), were included as covariates: “Fishing depth,” “Temperature at fishing,” “Wave exposure” (SWM), and “Salinity” (station level, *N* = 240, all VIF ≤ 2). When “Distance to open sea” was also considered, VIF values were 6.6 as a result of high correlation with “Salinity” (Pearson *r* = − 0.85, *N* = 240). Since “Salinity” provides a more direct relationship to explaining fish assemblage structure, it was selected for inclusion in the analyses, and potential aspects directly related to distance to the open are hence not covered (see also “Discussion” section).

There was also some level of correlation between salinity and variables representing eutrophication level. This was mainly seen in the hydrochemical monitoring data, which were not used in the GLMs (Pearson *r* = − 0.76 for total nitrogen, − 0.77 for chlorophyll *a*, and − 0.59 for Secchi depth, *N* = 56; Table S1). The correlation between ‘Salinity’ and ‘Secchi depth’ as collected in connection to the fish survey was lower (Pearson *r* = 0.44, *N* = 240). Compared to the other seven areas, the salinity in the no-take area was at the upper observed range (Fig. 2), while wave exposure was intermediate, and sampling depth stratification was identical to the other areas (Fig. 2, Table S1).

The covariates represent natural sources of variability, which might affect conclusions about other relationships of study unless controlled for (Bergström et al. 2016b; Östman et al. 2017b). The covariates were entered first, and a backwards stepwise selection procedure was applied to remove the least contributing variable at each step, based on the *t* values, until only terms with a significant (*p* < 0.05) contribution to the GLM were retained. After this, the relationship to water clarity was tested by ANOVA comparison of the retained model with a model also including “Secchi depth” (*N* = 240, *p* < 0.05). Subsequently, the contribution of “Area” (*N* = 8; fixed factor) was tested. Including “Area” as a fixed factor at this step was preferred over including it as a nested random factor in the baseline model, in order to evaluate any remaining differences among the specified areas of different character, after considering the covariates and “Secchi depth.” In cases where “Area” was included in the final model (*p* < 0.05), the final GLM was examined focusing on differences between the no-take area and the other seven areas.

The GLMs were run for the response variables *Total biomass*, *Total abundance* as well as the biomass of the trophic group *Piscivores* (defined by a trophic level ≥ 4.0; Froese and Pauly 2015), hereby including perch, northern pike and pikeperch, and the group *Non*-*piscivore*s, including all other species. The main taxonomic components of these groups were also assessed separately, namely, *Perch* and *Pikeperch* within the first group, as well as *Cyprinids* and *Other non*-*piscivores* within the latter. The taxonomic family Cyprinidae included roach, silver bream, bream, bleak, ide, rudd, tench, vimba, and crucian carp (for scientific names, see Table 2). Northern pike, which was also included in the piscivore group, was not assessed in separate due to infrequent occurrences giving poor model fit. Differences in fish sizes were estimated by the *Mean weight* and the abundance of large fish, the *Large perch indicator*, defined as the abundance of perch of at least 25 cm length. Mean weight was also assessed separately for the dominating species (*Mean weight of perch*), and all other species (*Mean weight of other species*). The abundance of large fish was only assessed with respect to perch, as other species were infrequent in this size class, so that estimates including all fish species did not provide additional information.

**Table 2:** Fish species recorded, their relative biomass and abundance as % of total catch, and presences in the studied areas. Species included in the group *Piscivores* are marked *, all others are categorized as *Non*-*piscivores*. Species included in the taxonomic family Cyprinidae are marked “c”

Name		Proportion		Presence							
English	Scientific	Biomass	Abundance	No-take	HighE1	HighE2	IM1	IM2	IM3	IM-R	LowE
Baltic herring	*Clupea harengus*	1.3	3.1	x	x	x	x	x	x	x	x
Baltic whitefish	*Coregonus maraena*	1.9	0.2	x	–	–	–	x	x	x	x
Black goby	*Gobius niger*	0.0	0.1	–	–	–	x	–	–	x	x
Bleak^c^	*Alburnus alburnus*	1.5	9.7	x	x	x	x	x	x	x	x
Bream^c^	*Abramis brama*	7.1	1.6	–	x	x	x	x	x	x	x
Bullhead	*Cottus gobio*	0.0	0.0	–	–	–	–	x	–	–	–
Crucian carp^c^	*Carassius carassius*	0.1	0.1	–	x	–	–	–	–	x	x
Eelpout	*Zoarces viviparus*	0.0	0.1	–	x	–	–	x	–	–	x
Flounder	*Platichthys flesus*	0.7	0.3	x	x	–	x	x	x	x	x
Four-horned sculpin	*Triglopsis quadricornis*	0.1	0.0	–	–	–	x	–	–	–	x
Greater sandeel	*Hyperoplus lanceolatus*	0.0	0.0	–	–	–	–	–	–	–	x
Ide^c^	*Leuciscus idus*	0.3	0.0	–	–	–	–	x	x	x	–
Northern pike*	*Esox lucius*	1.6	0.1	x	x	x	x	–	x	x	x
Perch*	*Perca fluviatilis*	45.7	26.6	x	x	x	x	x	x	x	x
Pikeperch*	*Sander lucioperca*	5.7	3.9	x	x	x	x	x	x	x	x
Roach^c^	*Rutilus rutilus*	20.8	30.4	x	x	x	x	x	x	x	x
Round goby	*Neogobius melanostomus*	0.0	0.0	–	x	–	–	–	–	–	–
Rudd^c^	*Scardinius erythrophthalmus*	0.0	0.0	–	–	–	–	–	–	x	–
Ruffe	*Gymnocephalus cernuus*	3.1	8.8	x	x	x	x	x	x	x	x
Silver bream^c^	*Blicca bjoerkna*	7.3	11.2	x	x	x	x	x	x	x	x
Smelt	*Osmerus eperlanus*	0.7	1.9	–	x	–	x	x	x	x	x
Sprat	*Sprattus sprattus*	0.4	1.5	x	x	x	x	x	x	x	x
Tench^c^	*Tinca tinca*	1.3	0.1	x	–	x	–	–	x	x	–
Three-spined stickleback	*Gasterosteus aculeatus*	0.0	0.1	–	–	–	–	–	–	x	–
Trout	*Salmo trutta*	0.4	0.0	–	–	–	–	–	–	–	x
Turbot	*Scophthalmus maximus*	0.0	0.0	–	–	–	–	–	–	–	x
Vimba^c^	*Vimba vimba*	0.1	0.1	–	–	–	x	–	x	x	–
Number of species		27		12	15	11	15	15	16	20	20

The GLMs also included two indicators applied in current status assessments of coastal fish in the Baltic Sea: the *Cyprinid indicator* and the *Piscivore indicator* (HELCOM 2018a, b). These indicators represent the number of fish above 11.0 cm length within each of the groups, defined as above. The *Cyprinid indicator* is expected to increase with eutrophication, whereas the *Piscivore indicator* has been associated with several pressure factors, including temperature and fishing (HELCOM 2018b).

The models were evaluated assuming a quasi-poisson distribution with a log link, except for metrics estimating mean weights for which a Gaussian distribution was used. Prior to the analyses, all station-wise environmental variables were evaluated for collinearity based on their variance inflation factors, which were ≤ 2.0 in all cases. The analyses were run using R3.0 as implemented in BRODGAR 3.4.7, Highland statistics (www.brodgar.com).

## Results

In total, this study reports 27 fish species (Table 2). The dominating species is perch, which constitutes 46% of all biomass. In all, piscivores represent 53% of the total biomass and 31% of the total abundance. Cyprinid taxa predominate among the non-piscivores, constituting 39% of the biomass and 53% of the total abundance.

### Species composition

The PCO based on data by stations explains slightly less than half of the total variation along the first two PCO-axes (46.7% for biomass and 47.4% for abundance; for additional PCO results, see Figs. S1–S2). The PERMANOVA based on data by station shows that there are strong overall differences in species composition among areas (Pseudo-F_7, 233_ = 12.4 for biomass, and 12.5 for abundances, both *p* < 0.001). In most cases, there are also significant pairwise differences between areas (*p* < 0.001, Fig. S3). The strongest differences in species composition occur between the no-take area and area HighE1, as well as between areas HighE1 and LowE (Fig. S3). The areas that are most similar to each other are IM-R, IM1, and IM3, as well as LowE and IM2 (0.01 < *p* < 0.05).

**Fig. 3:**
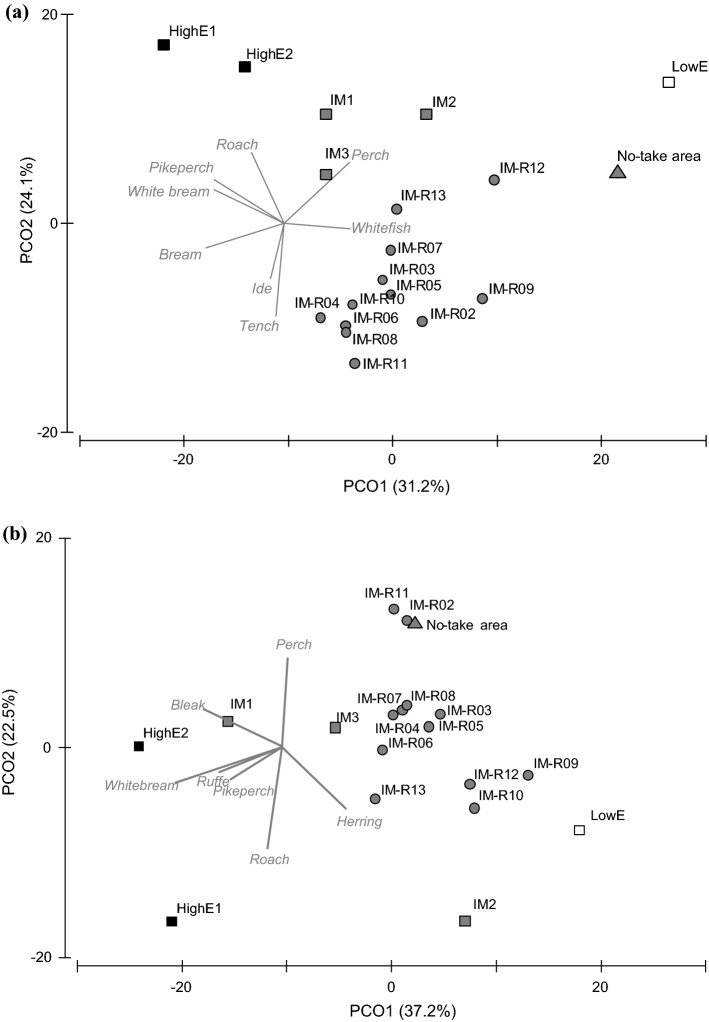
PCO biplot visualizing similarities in species composition among areas based on **a** biomasses and **b** abundance. Similarities are estimated by the Bray–Curtis index. Vectors show the correlation with the two first PCO-axes for species with a multiple correlation > 0.3

When the PCOs are re-run based on mean biomass values for each area, as confirmed by the station-wise PERMANOVA, the areas are clearly distributed along PCO1, which explains 31.2% of the total variation (Fig. 3a). Species contributing most to the observed pattern are on the one hand perch and whitefish (*Coregonus maraena*), which have highest biomasses in the no-take area as well as area LowE, and on the other hand bream, white bream, and pikeperch, which are relatively more dominating in areas with higher level of eutrophication. The variation among areas is generally higher than the variation among years for the long-term monitoring area IM-R, which is mainly reflected along PCO2. The corresponding analyses based on abundances explain a similar level of variation along the first two axes. The results mainly separate areas with higher level of eutrophication from the other areas (Fig. 3b), and are mostly explained by higher abundances of white bream, ruffe (*Gymnocephalus cernuus*), pikeperch, and bleak in the former areas. The no-take area is distinguished by higher abundances of perch and lower abundances of roach than most other areas. The species composition in the no-take area is similar to some years from the IM-R long-term data series, based on the first two PCO-axes (Fig. 3b), however these are separated along PCO3 (results not shown).

### Univariate analyses

The final GLMs explain on average 30% of the deviance in the data sets (range 12.0–53.5%; Table 3). Among the natural covariates, “Depth” is included in five of the models, reflecting an increase with depth for *Pikeperch*, *Other non*-*piscivores* (other than *Cyprinids*), and *Mean weight of other species* (other than perch), and a decrease with depth for the *Cyprinid indicator*. “Temperature” shows a positive relationship with metrics predominated by piscivores (*Total biomass*, *Piscivores*, and the *Piscivore indicator)*. The most frequently included covariate is “Wave exposure,” which is included in seven of the 14 final models. Several metrics predominated by non-piscivores increase with wave exposure (*Total abundance*, *Non*-*piscivores*, *Other non*-*piscivores*, and the *Cyprinid indicator*), while an inverse relationship is seen for *Piscivores* and *Large perch*. Five metrics decrease with increasing salinity (*Total abundance*, *Pikeperch*, *Non*-*piscivores*, *Cyprinids*, and the *Cyprinid indicator*), and one increases (*Other non*-*piscivores*).

**Table 3:** Summary of GLMs assessing the relation between univariate metrics and the covariates “Depth,” “Temperature,” “Wave exposure,” (SWM) and “Salinity,” as well as the relationship to ‘Secchi depth” and “Area”. The direction of the response is shown as increasing (+) or decreasing (−) according to the model, for factors significant at *p* < 0.01. As additional information, the direction of response for factors significant at *p* < 0.05 is shown as superscript. For effect of area, the signs indicate the relative difference between the concerned area and the no-take area. The last column shows the share of total deviance explained by each model

Response variable	Explanatory variables												Deviance explained (%)
	Covariates				Effect of Secchi depth	Effect of area							
	Depth	Temp.	SWM	Salinity		HighE1	HighE2	IM1	IM2	IM3	IM-R	LowE	
Abundance and biomass													
Total biomass	–	(+)		ns	–	ns	ns	(−)	ns	ns	(−)	ns	24
Total abundance	–	–	(+)	(−)	–	–	–	–	–	–	–	–	30.9
Biomasses per species groups													
Piscivores (all species)	–	(+)	(−)	–	–	ns^(−)^	(−)	(−)	ns	ns^(−)^	(−)	ns	32.5
Perch	–	ns^(+)^	ns^(−)^	ns	ns	(−)	ns	(−)	ns	ns^(−)^	(−)	ns	37.6
Pikeperch	(+)	–	–	(−)	(−)	–	–	–	–	–	–	–	25.1
Non-piscivores (all species)	–	–	(+)	(−)	(−)	–	–	–	–	–	–	–	25.7
Cyprinids	ns^(−)^	–	ns^(−)^	(−)	(−)	–	–	–	–	–	–	–	32
Other non-piscivores (than Cyprinids)	(+)	–	(+)	(+)	–	ns	ns	(−)	ns	ns^(−)^	ns^(−)^	ns	53.5
Fish sizes													
Mean weight (all fish)	–	–	(−)	–	–	(−)	(−)	(−)	(−)	(−)	(−)	(−)	31.8
Mean weight perch	–	–	ns	–	–	(−)	(−)	(−)	(−)	(−)	(−)	ns^(−)^	22
Mean weight other species (than perch)	(+)	–	–	–	–	ns	ns	(−)	ns	(−)	ns	ns	15.6
Large perch	–	ns	(−)	–	–	(−)	(−)	(−)	(−)	(−)	(−)	(−)	38.7
Indicators (abundance based)													
Cyprinid indicator	(−)	–	(+)	(−)	–	–	–	–	–	–	–	–	41.5
Piscivore indicator	–	(+)	–	–	–	ns	ns	ns	ns	ns	(−)	ns	12

**Fig. 4:**
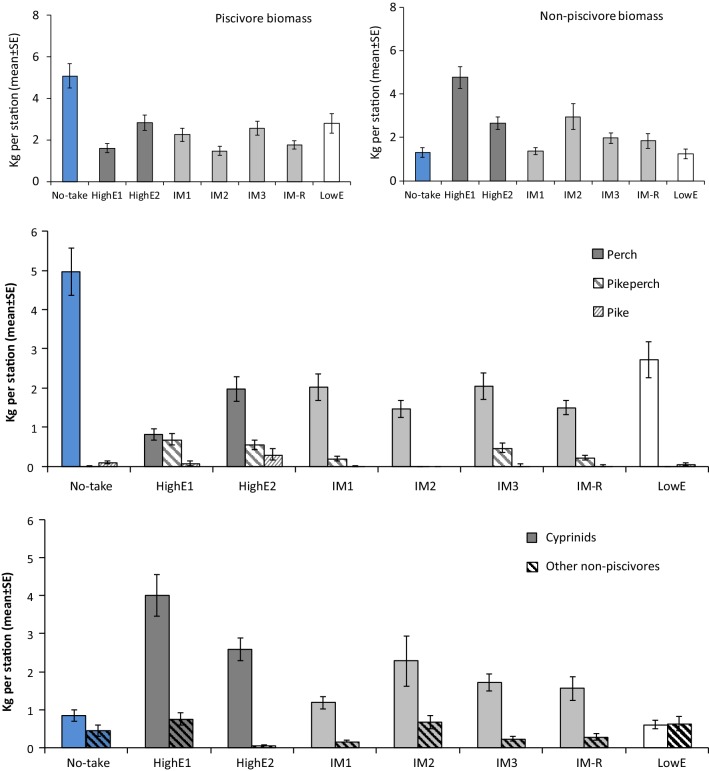
Mean catches of fish species groups and species per station in the no-take area (blue) and the other seven areas of different levels of eutrophication: HighE = highest level (dark gray), IM = intermediate (gray, and blue), LowE = lowest (white). Results are shown for biomasses *Piscivores* and *Non*-*piscivores,* as well as for the most common species and species groups within these

Three metrics, *Pikeperch*, *Non*-*piscivores,* and *Cyprinids* (all biomass based) show a relationship to “Secchi depth,” which was used as a proxy for level of eutrophication. In all three cases, increasing biomasses are seen at stations with poorer water clarity (lower “Secchi depth”). In the area with highest values for *Cyprinids* (HighE1), the total catches are 4.7 times higher than in the no-take area and 6.7 times higher than in the area with the lowest level of eutrophication (LowE). The areas categorized as most eutrophic also have the highest catches of pikeperch (Fig. 4).

**Fig. 5:**
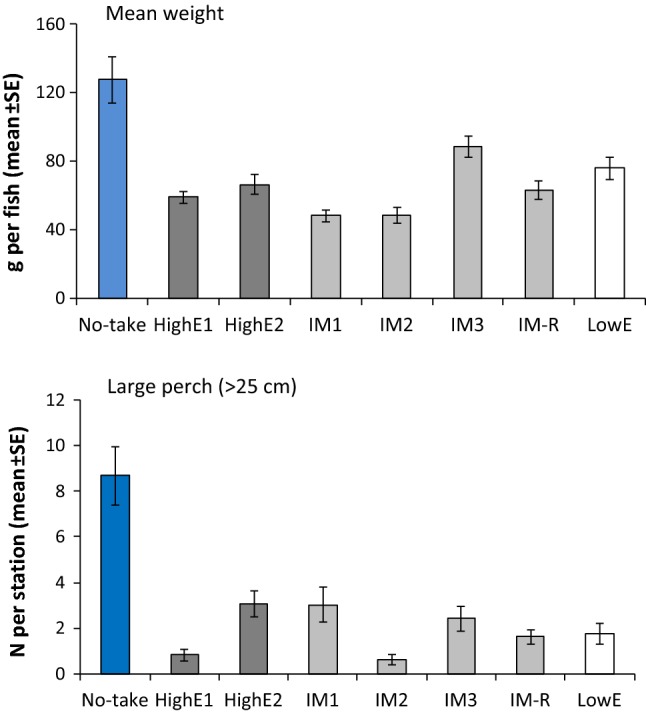
Values for two size-related metrics, *Mean weight* of fish and abundance of perch above 25 cm (*Large perch*), in the no-take area (blue) and the other seven areas of different levels of eutrophication: HighE = highest level (dark gray), IM = intermediate (gray, and blue), LowE = lowest (white)

An effect of “Area” is seen for nine of the metrics. In all these cases, values in the no-take area are higher than in at least some of the other areas (Table 3). Overall, the fish assemblage in the no-take area is most similar to that of the least eutrophic area (LowE), despite having clearly lower water clarity (“Secchi depth”; 2.2 ± 0.0 m in the no-take area, compared to 5.3 ± 0.1 m in LowE; Table S1). However, the two areas are distinguished by that fish *Mean weight* and the abundance-based *Large perch* are higher in the no-take area (Table 3, Fig. 5). *Mean weight* is higher in the no-take area than in all other areas, or 127 g in the no-take area compared to 48–88 g in the other areas. The difference is mainly attributed to perch (*Mean weight of perch,* see Table 3). Correspondingly, *Large perch* is also higher, with total catches in the no-take area being 3–11 times higher than in the other areas (Fig. 5).

**Fig. 6:**
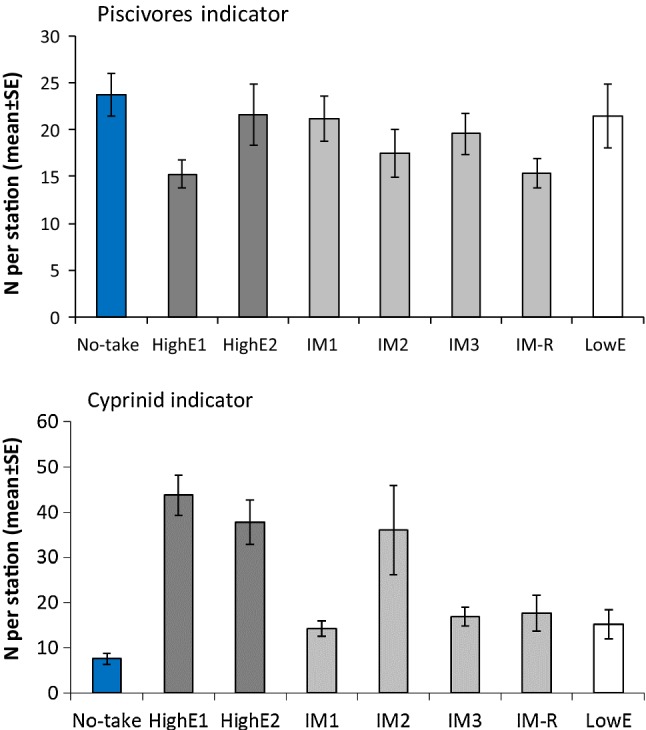
Values for the abundance-based *Piscivore indicator* and *Cyprinid indicator* in the no-take area (blue) and the other seven areas of different levels of eutrophication: HighE = highest level (dark gray), IM = intermediate (gray, and blue), LowE = lowest (white)

The overall biomass catch of *Piscivores* is 2–3 times higher in the no-take area compared to the other areas (Fig. 4). Due to these differences, combined with relatively lower catches of *Cyprinids*, the proportion of piscivores in relation to total fish biomass is 0.79 in the no-take area compared to between 0.25 and 0.69 in the other areas, or 1.2–3.2 times higher. With respect to abundances, these differences are less pronounced (Fig. 6).

Only one model, *Perch*, includes both “Secchi depth” and “Area.” This metric showed a positive relationship to “Secchi depth” before “Area” was included (*p* < 0.05, not in table), but not in the final model which also includes “Area.”

Two of the final models only include covariates, *Total abundance*, and the *Cyprinid indicator*. Additional analyses of these metrics show that they are negatively related to “Secchi depth if “Salinity” is not considered in the GLM. For the *Cyprinid indicator*, a model including “Depth,” “Wave exposure,” and “Secchi depth” shows a contribution from all these terms (*p* < 0.001), but the deviation explained is 29.7% compared to 41.4% for the model presented in Table 2. For *Total abundance*, a model including only “Wave exposure” and “Secchi depth” shows that both are significant contributors at *p* < 0.01, with an explained deviation of 21.4% compared to 30.9% in the main model (Table 2).

## Discussion

The study corroborates that no-take areas can enable an enhanced biomass and size structure of target species for fisheries (Lester et al. 2009; Fenberg et al. 2012), and, further, that they may contribute to improving the status of coastal fish in areas affected by eutrophication. The catch of piscivores in the no-take area exceeds observations in all other parts of the eutrophication gradient, where fishing is allowed, being 2–3 times higher in total and 2–7 times higher for the major piscivore, perch. The abundance of large perch, and accordingly fish mean size, is clearly higher in the no-take area than in the other areas, suggesting that perch size structure is truncated under ambient conditions, as a consequence of fishing.

The species composition in the no-take area is most similar to that of the area with lowest level of eutrophication, rather than having a species composition characteristic of similar, intermediate, eutrophication level. This suggests that the relatively stronger populations of piscivores may have regulated the biomass of non-piscivorous species, predominantly the Cyprinidae, thus giving rise to similar effects as those seen under conditions of low eutrophication, and in line with anticipated results from eutrophication abatement (HELCOM 2012).

Strong populations of piscivores, especially large piscivores, have also previously been connected to the enhancement of regulating ecosystem services, with non-piscivores (mesopredators) being associated with opposite features (Reiss et al. 2014; Östman et al. 2016). In the Baltic Sea, a regulating effect of perch and pike on three-spined stickleback has been observed, with indirect effects also on eutrophication-benefitted filamentous algae through a trophic cascade (Donadi et al. 2017). Similarly, examples of top-down regulation by perch and pike on Cyprinid species are evident from lakes, with effects cascading down to phytoplankton (see Jeppesen et al. 2012). Thus, it is not unlikely that various impacts on coastal ecosystems, usually attributed to responses to eutrophication, are reinforced by a high fishing pressure, although these aspects are not studied here (*c.f.* Eriksson et al. 2009, and others). In the current study, indications of top-down responses on mesopredators in the taxonomic group of Cyprinidae are observed. This group shows a conspicuous relationship to the studied environmental variables, including “Secchi depth” and “Salinity.” However, Cyprinid catches are lower in the no-take area compared to other areas with similar, intermediate, level of eutrophication, which might suggest regulation of this group by the abundant piscivore populations.

In this context, the results also serve as an illustration of potentially lost ecosystem services and values under current management. Even though piscivorous fish are appreciated for fishing, both for food provision and sports fishing, the results corroborate that improved fisheries management has a potential for substantial gain also for other ecosystem services.

In order to strengthen fish populations, many different management measures may be used, including gear or catch restrictions, in addition to seasonal or year-round closures. No-take areas have mainly proven efficient for restoring fish populations within the closed areas (Lester et al. 2009), but their effects may also spill over to adjacent areas in the form of juvenile or adult fish (Halpern et al. 2010; Pelc et al. 2010). Indirect effects may also occur, through protecting populations against evolutionary effects of size-selective fishing, as well as against population collapses connected to management failure (Baskett and Barnett 2015).

Given the limited spatial distribution of the current no-take area, and that it is the only one present in the studied coastal region, however, such wider-scale effects are expected to be rather limited here. The lack of comparable areas along the coast also entailed a weakness to the study, as it was not possible to replicate the no-take area in the analyses. A limited number of other no-take areas do exist in Sweden, but these have been in place for a much shorter period of time, are located farther away and have different environmental settings (Bergström et al. 2016c). Therefore, it was not possible to include them in the same analytical framework. Over time, to enable a more stringent evaluation, more no-take areas need to be implemented. Such a management direction is also supported by the potential benefits indicated here. In this respect, an additional ecosystem service of the no-take area is its value as a reference area for science and management. No-take areas provide a rare opportunity to isolate the effects of fishing from other pressures acting on the marine environment, and are thus central for increasing our understanding of human impacts on coastal ecosystems.

In the present situation, the replication challenge was met by ensuring a wide range of environmental variability in the areas that were used for comparison, and by applying a semi-descriptive approach to the GLM. First, response variables were related to natural environmental variables, and then any remaining variability was explored in relation to differences in the level of eutrophication. The last step focused on comparing the no-take area with the other areas, after accounting for other environmental variables. This setup provides a relatively conservative test, even though several sources of remaining variation are still not included. For example, differences in habitat suitability are partly captured by the covariates (Kallasvuo et al. 2016), but could also contribute to remaining differences among areas. Another factor that may have contributed is the distance to open sea, which was highly correlated to salinity in this study, and therefore not included specifically. Proximity to open sea has previously been shown to have an influence on local fish assemblages through enhanced biotic interaction with migrating species, such as the three-spined stickleback (Bergström et al. 2015; Donadi et al. 2017).

The results regarding metrics on piscivore biomass and fish sizes are, however, not as likely explained by other mechanisms than by the fishing closure (see also Olin et al. 2017). Other pressures that might influence these results are noise and physical disturbance from boating, which is forbidden in the no-take area. Absence of boating can be expected to additionally improve the conditions for fish, particularly for early life stages (Sandström et al. 2005; Hansen et al. 2018). However, only small fractions of the other study areas had jetty densities that can be expected to give rise to negative effects on fish recruitment, why this potential effect is expected to be negligible compared to fishing. Regarding mortality factors, natural predation from cormorants and seals could provide an impact (Vetemaa et al. 2010; Hansson et al. 2017). Both cormorants and gray seal occur in the studied areas, but due to their wide foraging ranges, reliable estimates at the level of each study area were not available in order to consider this aspect in the environmental categorization of areas. A selective preference for certain feeding areas can still not be excluded, and may contribute to some of the variation among areas identified in the GLMs.

The results also show the importance of considering species habitat preferences in spatial management. The main results of the study are largely driven by perch, which is a dominating species in the current coastal area, whereas catches of the second most common piscivore, pikeperch, are predominantly related to decreased water clarity. Pikeperch catches are overall scarce in the data, and due to the low occurrences it was not possible to evaluate the results specifically with regard to size structure. However, with respect to total biomass, pikeperch is most common in the two areas with highest level of eutrophication, and it is not favored in the no-take area. These results reflect the natural habitat preferences, as pikeperch gains from the turbid conditions caused by excessive nutrient loading (Bergström et al. 2013). The third major piscivore of the system, pike, is not representatively caught by gillnets used in this study. Earlier studies have, however, shown that pike biomasses, like for perch, are clearly higher in the no-take area (see Bergström et al. 2016c).

Further, with respect to the eutrophication gradient, relatively higher catches of the species within the taxonomic family of Cyprinidae were expected a priori in the areas with more nutrient-rich conditions (HELCOM 2018b). The obtained results support the expectations with respect to the biomass of Cyprinids, but not with respect to their abundance, which is only related to natural environmental variables including salinity (Table 3, Fig. 6). In this regard, the results suggest that the abundance-based *Cyprinid indicator* may not be sensitive enough to eutrophication when assessed in a salinity gradient, while the biomass-based metric *Cyprinids* can be more useful.

In all, four out of five metrics showing an inverse relationship with salinity can in fact be explained in the light of variation in Cyprinid species (*Total abundance, Non*-*piscivores, Cyprinids,* and the *Cyprinid indicator*), likely reflecting that Cyprinid species can be restricted by the availability of low salinity areas for recruitment in the Baltic Sea (Härmä et al. 2008). Even though the correlation between the variables “Secchi depth” and “Salinity” was low enough to include them in the same model in this study, the similar direction of results from alternative models including “Secchi depth” instead of “Salinity” illustrates the difficulty of separating effects from co-occurring environmental variables in empirical studies. It is also important to note that water clarity, measured by the Secchi depth, encompasses only part of the effects of eutrophication. Differences in water clarity are expected to affect fish directly, influencing the relative reproductive output and feeding efficiency of different species (*cf* Sandström and Karås 2002; Bergström et al. 2013), while other variables used for environmental characterization (Table 1) provide information on the general level of eutrophication. Secchi depth may, in turn, also be affected by species composition in the fish assemblage, as this may influence on the level of grazing on phytoplankton through trophic cascades (Jeppesen et al. 2012).

Overall, our study suggests that both fishing and eutrophication have strong effects on coastal fish assemblages, and that their effects may to some extent be similar, such as decreasing the proportion of piscivores and increasing that of mesopredators (predominantly Cyprinidae), so that the effects of these pressures are most likely to accentuate each other in the ecosystem. Similar synergistic interaction effects of eutrophication and fishing have previously been demonstrated experimentally in the Baltic Sea (Eriksson et al. 2009; Sieben et al. 2011), strongly supporting the conclusions of the current field study.

## Conclusions

This study illustrates how spatial variability in coastal fish assemblages can be shaped by local variation in natural variables, together with variation in level of eutrophication and differences in fishing pressure, influencing both species composition and size structure.

Most noticeably, *Perch* is benefitted in the no-take area, reaching a larger size than in the other areas, which is also reflected in elevated mean weight and biomass.

The *Cyprinid* biomass is benefitted by eutrophication, showing higher values in areas of higher eutrophication level, and an inverse relationship to water clarity. Further, Cyprinid biomass in the no-take area is lower than could be expected given its intermediate level of eutrophication, suggesting a top-down effect from predation.

*Pikeperch* prefers areas with higher level of eutrophication, and is not benefitted by the no-take area. However, pikeperch is generally scarce. This exemplifies the importance of considering species-specific preferences in habitat protection, and ensuring protection of a range of different habitat types.

Among established environmental indicators used in the Baltic Sea (HELCOM 2012), the results suggest that the abundance-based *Cyprinid indicator* may not perform well for comparing areas with strong differences in salinity, but that the corresponding biomass-based indicator may be more useful. The abundance-based *Piscivore indicator* may not be sensitive enough to changes in fishing pressure and could be supplemented by an indicator on large fish.

The metric *Large perch* shows substantially higher values in the no-take area compared to the other areas, suggesting that the abundance of this main piscivore is currently far from its potential in the studied coastal system.

## Electronic supplementary material

Below is the link to the electronic supplementary material.

Supplementary material 1 (PDF 633 kb)
