# Supplementary material for: Relative impacts of fishing and eutrophication on coastal fish assessed by comparing a no-take area with an environmental gradient
Source: Ambio. 2018 Dec 6;48(6):565–79. doi: 10.1007/s13280-018-1133-9 (PMC6486898; doi:10.1007/s13280-018-1133-9)
Supplement: Supplementary file 1 — Supplementary material 1 (PDF 633 kb) [file 13280_2018_1133_MOESM1_ESM.pdf]

Ambio

Electronic Supplementary Material

Title: **Relative impacts of fishing and eutrophication on coastal fish assessed by comparing a no-take area with an environmental gradient**

Authors: Lena Bergström, Martin Karlsson, Ulf Bergström, Leif Pihl, Patrik Kraufvelin

Supporting information

S1: Principal coordinates analysis of similarities in species composition among stations and outcome of PERMANOVA comparison

Similarities in species composition among stations in the eight studied areas were estimated by the Bray-Curtis index and principal coordinated analyses (PCO). The analyses were run separately with respect to catches of fish biomass, and abundances (Fig S1-2).

For biomasses, the first axis is mainly attributed to variation in perch (*Perca fluviatilis*), pikeperch (*Sander lucioperca*) and silver bream (*Blicca bjoerkna*), whereas the second axis is mainly related to herring (*Clupea harengus*) and smelt (*Osmerus eperlanus*). For abundances, the first axis mainly represents variation in roach (*Rutilus rutilus*) and herring, and the second axis mainly represented variation in ruffe (*Gymnocephalus cernuus*), pikeperch and silver bream.

For the PCO based on biomasses, the first PCO-axis (PCO1) explains 26.7 % of the total variation in the data set, PCO2 explains 20.0 % and PCO3 11.9% For the PCO based on abundances, the corresponding results are 26.0% for PCO1, 21.5% for PCO2, and 17.4% for PCO3.

A detailed account of environmental information for characterizing the study areas in relation to eutrophic level, and other variables (salinity, temperature at fishing, depth at fishing, and wave exposure), is provided in Table S1.

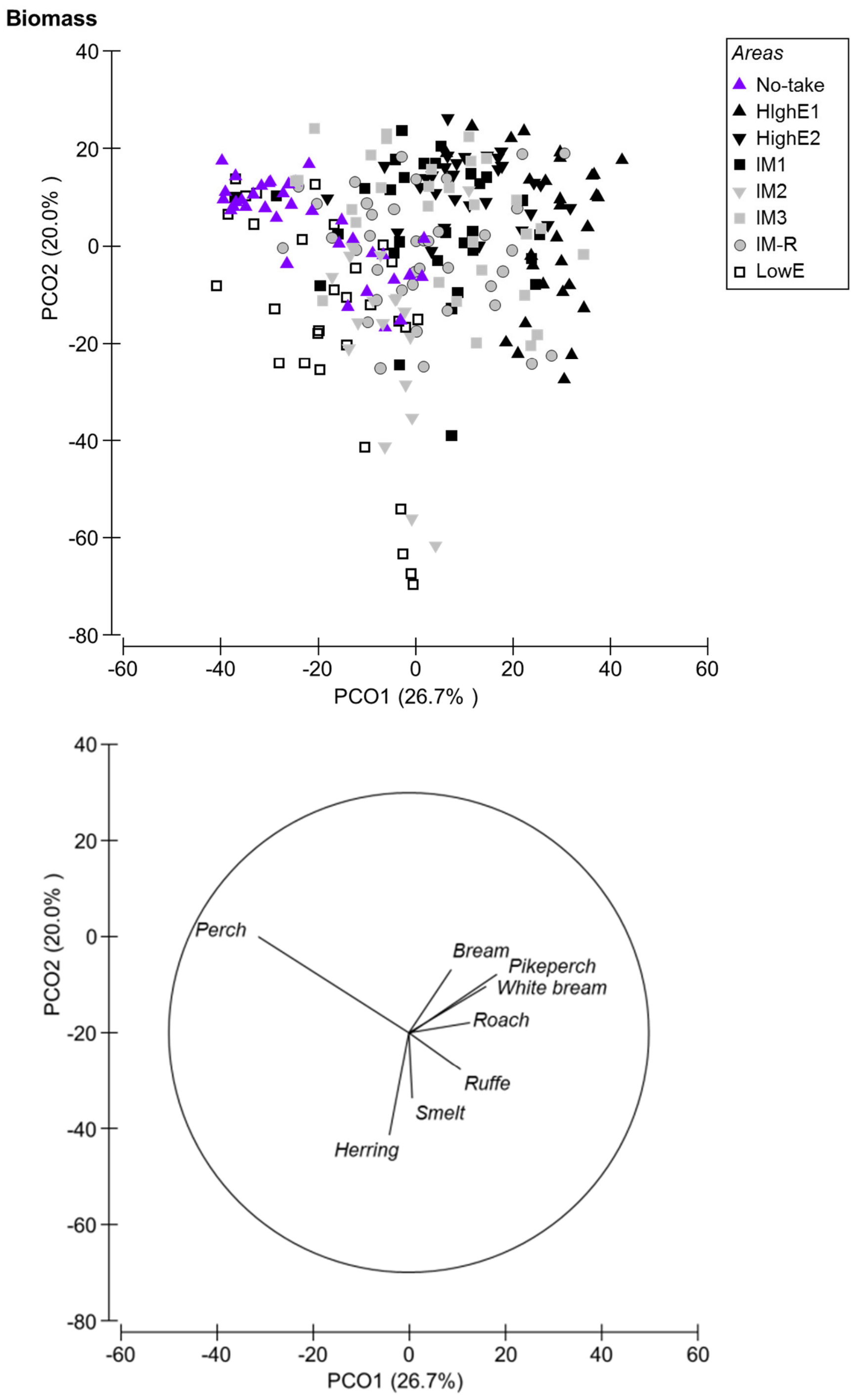

Figure S1. PCO biplot showing similarities in species composition among stations, based on biomasses. Vectors plots (lower picture) show the correlation with the first and the second PCO axes, for species with a multiple correlation >0.3 (perch: *Perca fluviatilis*, bream: *Abramis brama*, pikeperch: *Sander lucioperca*, white bream: *Blicca bjoerkna*, roach: *Rutilus rutilus*, ruffe: *Gymnocephalus cernuus*, smelt: *Osmerus eperlanus*, herring: *Clupea harengus*).

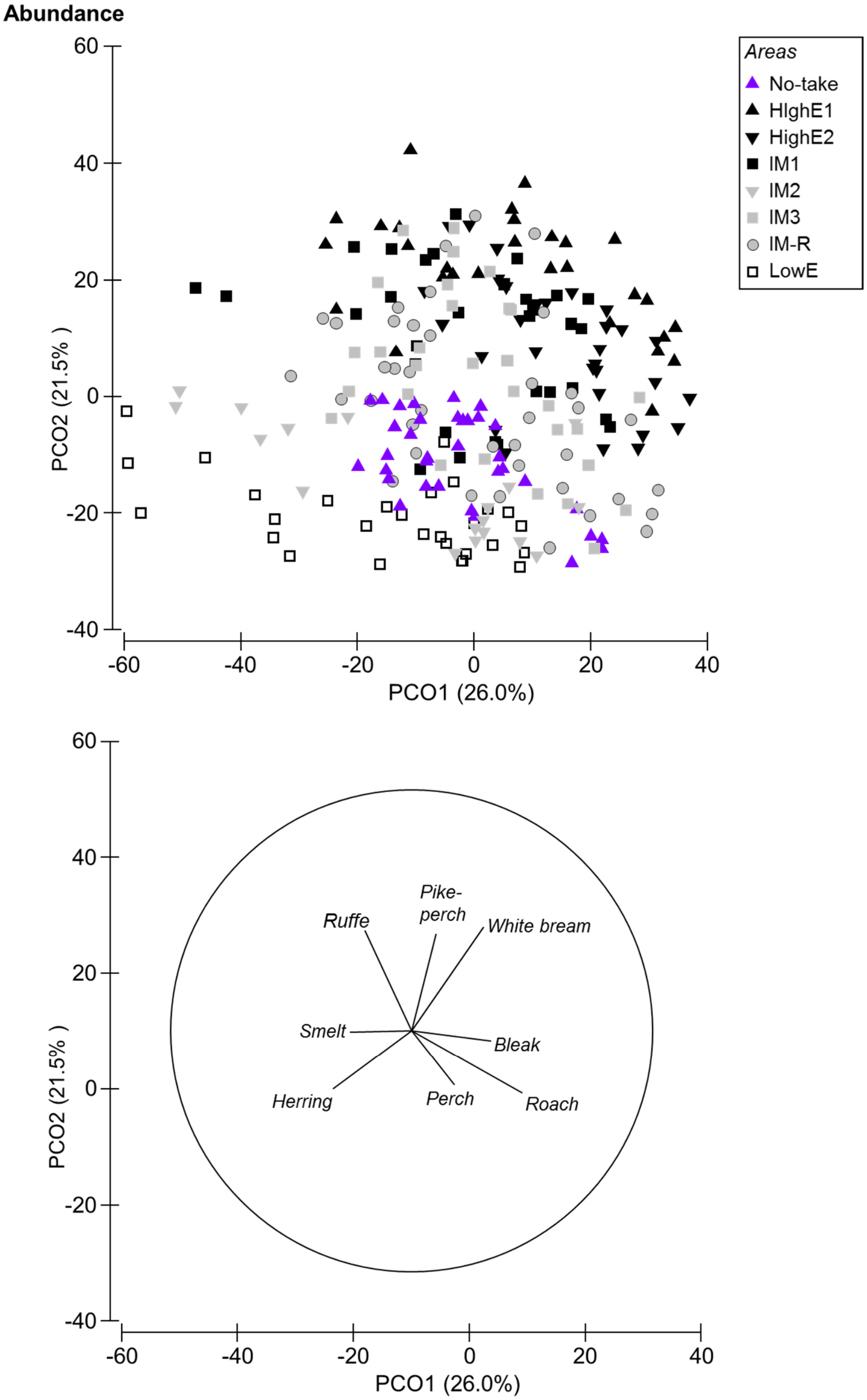

Figure S2. PCO biplot showing similarities in species composition among stations, based on abundance data. Vectors plots (lower picture) show the correlation with the first and the second PCO axes, for species with a multiple correlation >0.3 (ruffe: *Gymnocephalus cernuus,* pikeperch: *Sander lucioperca*, white bream: *Blicca bjoerkna,* bleak: *Alburnus alburnus*, roach: *Rutilus rutilus,* perch: *Perca fluviatilis*, herring: *Clupea harengus,* smelt: *Osmerus eperlanus*).

Figure S3. Pairwise comparison of areas with respect to similarity in species composition, based on biomasses (left) and abundances (right) and the Bray-Curtis index. Higher t-values indicate stronger differences, based on PERMANOVA (dark bars: $p < 0.001$, white bars: $0.01 < p < 0.05$).

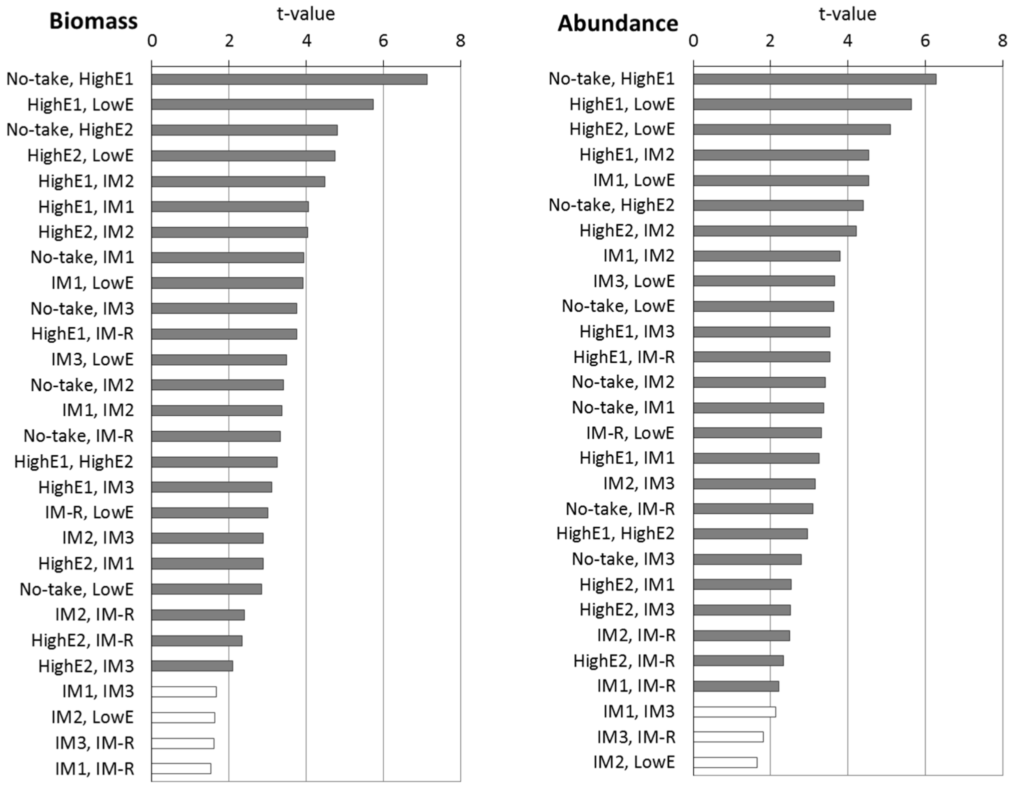

Table S1. Environmental characterization of the studied areas. The letters identify discrete groups based on *a posteriori* SNK-tests, for variables with significant overall differences according to ANOVA ($p<0.01$). In these cases, F-ratios are shown in the last column1 (ns = not significant). D.f. = 6, 56 for variables based on hydro-chemical monitoring data, and d.f. =7, 232 when measured during test fishing. Values show the mean ± SE (na = not available). For location of subareas, see Fig. 1.

**Variable**	**No-take area**	**HighE1**	**HighE2**	**IM1**	**IM2**	**IM3**	**IM-R**	**LowE**	**ANOVA F**
Eutrophic level									
Total nitrogen, monitoring (µM)	na	29.1 ±1.4 b	39 ±1.7 a	27.4 ±0.8 bc	24.6 ±0.4 c	27.3 ±0.6 bc	26.5 ±0.5 bc	24.1 ±0.7 c	26.4
Total phosphorus, monitoring (µM)	na	0.77 ±0.1	0.84 ±0.1	0.76 ±0.0	0.68 ±0.0	0.75 ±0.0	0.75 ±0.0	0.69 ±0.0	Ns
Chlorophyll-*a*, monitoring (µgl^{-1})	na	10 ±1.6 a	9.3 ±0.8 a	3.7 ±0.6 c	5.5 ±0.4 b	3.5 ±0.4 c	3.4 ±0.3 c	3.2 ±0.6 c	16.6
Water clarity, monitoring (Secchi depth, m)	na	2.0 ±0.3 a	2.0 ±0.1 a	2.7 ±0.3 ab	1.8 ±0.2 a	3.1 ±0.2 b	3.2 ±0.3 b	5.2 ±0.5 c	15.9
Water clarity at fishing station (Secchi depth, m)	2.2 ±0.0 c	0.8 ±0.1 a	1.9 ±0.1 b	2.3 ±0.1 c	3.0 ±0.1 d	2.8 ±0.1 d	2.2 ±0.1 c	**5.3** ±0.1 e	351
Other									
Salinity (Jun-Aug)	na	4.3 ±0.2 b	3.3 ±0.1 a	5.7 ±0.2 d	4.7 ±0.1 c	5.6 ±0.1 d	6.1 ±0.1 d	6.2 ±0.0 d	61.6
Salinity at fishing station	6.5 ±0.0 g	4.7 ±0.1 b	4.0 ±0.0 a	6.2 ±0.0 e	5.5 ±0.1 c	6.0 ±0.0 d	6.4 ±0.0 fg	6.3 ±0 ef	399
Distance to baseline from fishing station (km)	15.0 ±0.14 a	39.3 ±0.38 b	52.2 ±0.33 c	18.6 ±0.22 d	25.1 ±0.48 e	27.1 ±0.33 f	13.2 ±0.26 g	13.2 ±0.41 g	2,144
Wave exposure (SWM, log) at fishing station	3.6 ±0.0 c	4.1 ±0.0 a	3.7 ±0.0 c	3.6 ±0.0 c	4.0 ±0.1 ab	3.6 ±0.0 c	3.6 ±0.1 c	3.9 ±0.1 b	16.2
Temperature at fishing station (°C)	19.1 ±0.1 a	16.6 ±0.3 b	18.5 ±0.2 a	18.3 ±0.4 a	14.3 ±0.5 c	16.7 ±0.3 b	19 ±0.3 a	16.1 ±0.4 b	24.1

Depth at fishing station (m)	5.3 ±0.4	5.1 ±0.5	5.0 ±0.4	5.4 ±0.5	5.5 ±0.7	5.4 ±0.5	4.8 ±0.3	5.3 ±0.5	Ns
